# VDAC1‐Targeted NHK1 Peptide Recovers Mitochondrial Dysfunction Counteracting Amyloid‐β Oligomers Toxicity in Alzheimer's Disease

**DOI:** 10.1111/acel.70069

**Published:** 2025-04-13

**Authors:** Fabrizio Cavallaro, Stefano Conti Nibali, Salvatore Antonio Maria Cubisino, Pietro Caruso, Stefania Zimbone, Iolanda Rita Infantino, Simona Reina, Vito De Pinto, Angela Messina, Maria Laura Giuffrida, Andrea Magrì

**Affiliations:** ^1^ Department of Biomedical and Biotechnological Sciences University of Catania Catania Italy; ^2^ Institute of Crystallography National Research Council (CNR‐IC) Catania Italy; ^3^ Department of Biological, Geological, Environmental Sciences University of Catania Catania Italy

**Keywords:** Alzheimer's disease, amyloid‐β, interfering peptide, mitochondria, VDAC1

## Abstract

Mitochondrial dysfunction has been implicated in a broad range of age‐related pathologies and has been proposed as a causative factor in Alzheimer's disease (AD). Analysis of post‐mortem brains from AD patients showed increased levels of Voltage‐dependent anion‐selective channel 1 (VDAC1) in the dystrophic neurites surrounding amyloid‐β (Aβ) deposits, suggesting a direct association between VDAC1 and mitochondrial toxicity. VDAC1 is the most abundant pore‐forming protein of the outer mitochondrial membrane and, as a channel, it plays a pivotal role in regulating cellular bioenergetics, allowing the continuous exchange of ions and metabolites (ATP/ADP, Krebs cycle intermediates) between cytosol and mitochondria. In light of this evidence, we looked into the effects of Aβ oligomers on VDAC1 functions through electrophysiological and respirometric techniques. Our findings indicate that Aβ oligomers significantly modify the conductance, voltage dependency, and kinetic features of VDAC1, as well as its slight selectivity for anions, leading to a marked preference for cations. Given that VDAC1 is mainly involved in the trafficking of charged molecules in and out of mitochondria, a general reduction of cell viability and mitochondrial respiration was detected in neuroblastoma cells and primary cortical neurons exposed to Aβ oligomers. Interestingly, the toxic effect mediated by Aβ oligomers was counteracted by the use of NHK1, a small synthetic, cell‐penetrating peptide that binds and modulates VDAC1. On these results, VDAC1 emerges as a crucial molecule in mitochondrial dysfunction in AD and as a promising pharmacological target for the development of new therapeutic avenues for this devastating neurodegenerative disease still without a cure.

## Graphical abstract

Amyloid‐β (Aβ) oligomers affect VDAC1 electrophysiology and the physiological metabolic cross‐talk between cytosol and mitochondria. The use of a small, synthetic VDAC1‐antagonist peptide, derived from hexokinase, restores mitochondrial functionality, and respiration in cellular models of Alzheimer's disease.

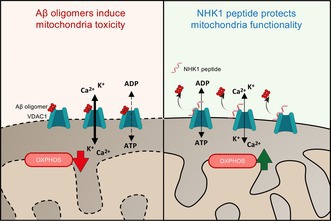

## 1 Introduction

Aging represents the greatest risk factor for Alzheimer's disease (AD), the most diffused form of dementia in the elderly (Guerreiro and Bras 2015). The progressive decline of cognitive abilities and memory typical of AD correlates, at the molecular level, with the deposition of amyloid‐β (Aβ) peptide and the hyperphosphorylation of Tau (p‐Tau) protein within the brain (Knopman et al. 2021).

Aβ peptides, consisting of 40 (Aβ_1–40_) or 42 (Aβ_1–42_) amino acids, are generated from the sequential cleavage by β‐ and γ‐secretases of the Amyloid Precursor Protein (APP_695_), an integral protein that principally enriches the plasma membranes of neuronal synapses but also localizes in various intracellular compartments (Nunan and Small 2000; Thinakaran and Koo 2008; Xu et al. 1997; Hartmann et al. 1997). As it is highly hydrophobic, Aβ_1–42_ is particularly prone to self‐assembly into multimeric forms, mainly fibrils—the key component of extracellular amyloid plaques—and intermediate products of low molecular weight, such as oligomers and protofibrils (Chen et al. 2017).

Among different species, Aβ oligomers are regarded as highly neurotoxic, being able to accumulate also within the neurons, diffuse from one cell to another, and serve as seeds for aggregation (LaFerla et al. 2007; Gouras et al. 2000; Sardar Sinha et al. 2018; Domert et al. 2014). Intracellularly, Aβ oligomers may localize in mitochondria, where they are believed to be responsible for interfering with several activities and enzymes, thus promoting organelle dysfunction (Manczak et al. 2006; Pinho et al. 2014; Hernandez‐Zimbron et al. 2012; Lustbader et al. 2004; Takuma et al. 2005). Mitochondria are of pivotal importance in regulating cellular bioenergetics, being mainly involved in the production of ATP via oxidative phosphorylation, as well as in the regulation of phospholipid biosynthesis, calcium homeostasis, and apoptosis. Considering that the human brain accounts for about 20% of the body's total energy requirement, it is not surprising that mitochondrial dysfunction is largely considered an early and crucial event in the onset and progression of AD that strictly contributes to the decrease of cell metabolism, synaptic failure, and neuronal loss (Mink et al. 1981; Bhatia et al. 2022).

On the outer membrane of mitochondria (OMM), the Voltage‐dependent anion‐selective channel 1 (VDAC1), also known as mitochondrial porin, emerges as the most abundant, ubiquitously expressed, and evolutionary conserved isoform of a three‐member family of pore‐forming proteins (Messina et al. 2012). VDAC1 is substantially a β‐barrel made of 19 antiparallel β‐strands, with the exception of the N‐terminal domain, structured as α‐helix and located in the pore lumen (Hiller et al. 2008; Ujwal et al. 2008; Manzo et al. 2018). Given its pivotal location at the interface between the cytosol and the mitochondrion, VDAC1 principally regulates the metabolic cross‐talk between the two compartments, allowing the passive diffusion of ions (Mg^2+^, Na^+^, Cl^−^, Ca^2+^), small metabolites under 5 kDa (pyruvate, succinate, malate), NAD^+^/NADH, and ATP/ADP (Rostovtseva and Colombini 1997; Hodge and Colombini 1997; Colombini 2004).

In physiological conditions, VDAC1 interacts with many cytosolic proteins, including Hexokinases (HKs) and specific members of the Bcl‐2 family (Bak, Bax), thus participating in the regulation of apoptosis (Tsujimoto and Shimizu 2000; Anflous‐Pharayra et al. 2007; Shoshan‐Barmatz et al. 2009). On the other hand, in AD tissues VDAC1 has been identified as the preferential mitochondrial binding site for intracellular Aβ peptides, full‐length APP, and Tau (Manczak and Reddy 2012; Reddy 2013). The affinity of Aβ for VDAC1 was confirmed by chemical–physical techniques and electrophysiology. Precisely, the addition of Aβ aggregates to VDAC1 reconstituted in artificial membranes induced an increase in channel conductance that might promote the porin oligomerization and, in turn, the activation of apoptosis (Smilansky et al. 2015). However, the involvement of VDAC1 in AD is much more extensive.

A progressive, age‐related increase in the expression levels of VDAC1 was observed in cortical tissues from post‐mortem brains of AD patients and APP transgenic mice (Cuadrado‐Tejedor et al. 2011; Manczak and Reddy 2012). On the contrary, reduced levels of VDAC1, as observed in VDAC1^+/−^ mice, correlated with a decrease in the expression levels of the AD‐related genes APP, Tau, PS1, PS2, and BACE1 (Manczak et al. 2013). Consistently, the reduction of VDAC1 expression, along with APP and Tau genes by RNA silencing, significantly improved both mitochondrial and synaptic activity, with beneficial effects on oxidative stress and ATP production (Manczak and Reddy 2013). Similarly, the partial reduction of VDAC1 expression in AD transgenic mice counteracted Tau‐related pathology by increasing mitochondrial fission and biogenesis, mitophagy, and synaptic functions (Vijayan and Reddy 2022; Vijayan et al. 2022). Overall, these data underline the importance of Aβ‐Tau‐VDAC1 interplay for AD onset and progression.

With this in mind, here we focused on the impact of Aβ_1–42_ oligomers on VDAC1 electrophysiology and mitochondrial respiration. Our results indicate that Aβ oligomers profoundly alter the physiological behavior of VDAC1 channels by affecting the conductance, voltage dependency, opening/closing kinetics, and selective permeability of ions, including calcium. Furthermore, the addition of Aβ oligomers to permeabilized neuroblastoma cells or intact primary cortical neurons considerably diminished the mitochondrial respiration associated with the porin activity. Remarkably, the detrimental outcome of Aβ oligomers was prevented by the addition of NHK1, a small interfering peptide capable of binding VDAC1. Overall, our results point to a key role of VDAC1 in activating the molecular cascade leading to the mitochondrial dysfunction in AD and provide new insights for the development of a pharmacological therapy based on VDAC1 modulation.

## 2 Results

### 2.1 Aβ Oligomers Affect the Channel Conductance of VDAC1 Reconstituted in Artificial Membranes

The interaction between human VDAC1 and Aβ oligomers was assayed at the planar lipid bilayer (PLB) by using the recombinant protein expressed in *Escherichia coli* and purified by affinity chromatography (Figure S1A). Since the heterologous expression of membrane proteins in bacterial systems leads to the loss of proper conformation and activity, the purified VDAC1 was refolded by dialysis. To avoid the presence of aggregates, a size‐exclusion chromatography was performed (Figure S1B), and only the elution fraction containing the monomeric form was used for further experiments (Figure S1C).

Aβ oligomers were prepared from synthetic Aβ_1–42_ according to a well‐established protocol, known to produce small, soluble, globular oligomeric species which represent the primary toxic form among Aβ aggregates (Lambert et al. 1998). Characterization of Aβ aggregates and freshly prepared monomers used as controls has been conducted by Western blot analysis (Figure S2). To exclude any direct effect of Aβ on the artificial membrane, oligomer solution was added to both *cis* and *trans* sides of the phospholipid bilayer at the final concentration of 0.3 μM. As displayed in Figure S3, no deviations from the baseline current (0 pA) were recorded upon application of a triangular voltage wave of ±50 mV, suggesting that the membrane integrity was unaffected throughout the entire analysis.

**FIGURE 1:**
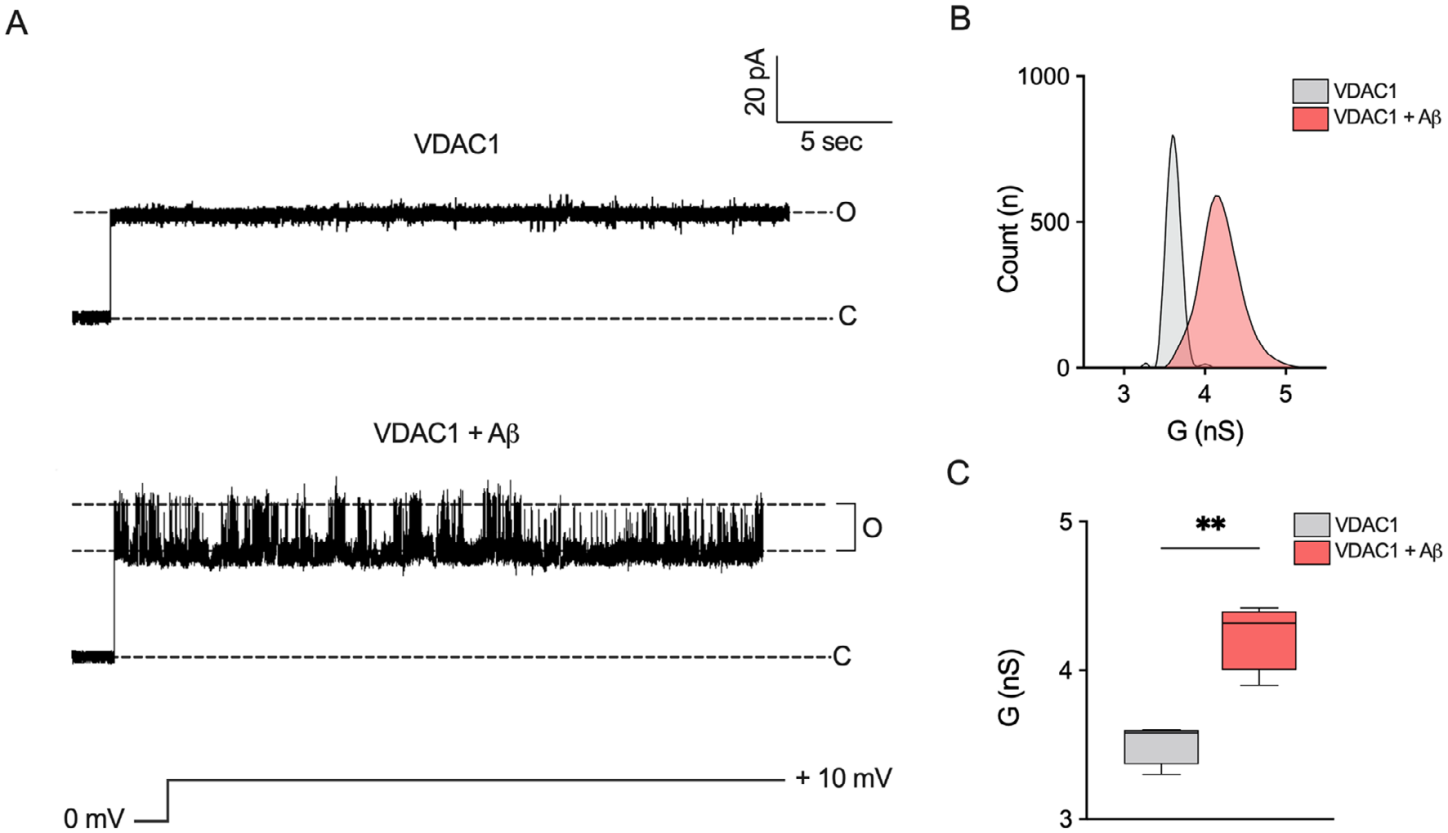
Aβ oligomers modify the channel conductance of VDAC1 at the Planar Lipid Bilayer. (A) Representative current traces of single‐channel analysis of reconstituted VDAC1 in artificial membranes before (upper trace) and after the addition of Aβ oligomers at the final concentration of 0.3 μM (lower trace). Traces were recorded at the constant voltage of +10 mV in symmetrical 1 M KCl buffer. O, open state; C, closed state. A *n* = 4 independent experiments were performed. (B, C) Quantitative analysis of VDAC1 conductance frequency (B) and average conductance (C) as a function of the events relative to the traces displayed in (A). Data in (C) are expressed as a mean ± SEM of *n* = 4 independent experiments and statistically analyzed by unpaired *t* test, with **p < 0.01.

Conductance (G) of VDAC1 was then investigated before and after the addition of Aβ oligomers at the PLB in a symmetric 1 M KCl with a constant applied voltage of +10 mV. As shown in the upper trace of Figure 1A, VDAC1 stably persisted in a high‐conducting or open state (O), as expected in these experimental conditions. The addition of 0.3 μM Aβ oligomers to both sides of the bilayer induced fast and reversible events characterized by higher amplitude (Figure 1A, lower trace), as well as a shift in the frequency distribution and average conductance toward higher values (Figure 1B,C). In agreement with the literature, VDAC1 displayed an average value of *G* = 3.52 ± 0.14 nS (Benz et al. 1989; Magrì et al. 2018), while the addition of Aβ oligomers to the system caused a significant increase in conductance (*G* = 4.24 ± 0.23 nS, *p* = 0.0018, Figure 1C). Overall, these data indicate that Aβ oligomers modify the conductance of reconstituted VDAC1 in artificial membranes.

### 2.2 Aβ Oligomers Reduce the Responsivity of VDAC1 to the Applied Voltage

It is well known that VDAC1 conductance varies as a function of the voltage applied. Below ±20–30 mV, the current amplitude of mitochondrial porin increases linearly with the voltage, and the channel stably persists in a high‐conducting state, the so‐called open state. Conversely, the application of potentials higher than ±30 mV induces a step‐like transition toward the low‐conducting state(s) or closed state(s) (Zachariae et al. 2012; Magrì et al. 2018).

**FIGURE 2:**
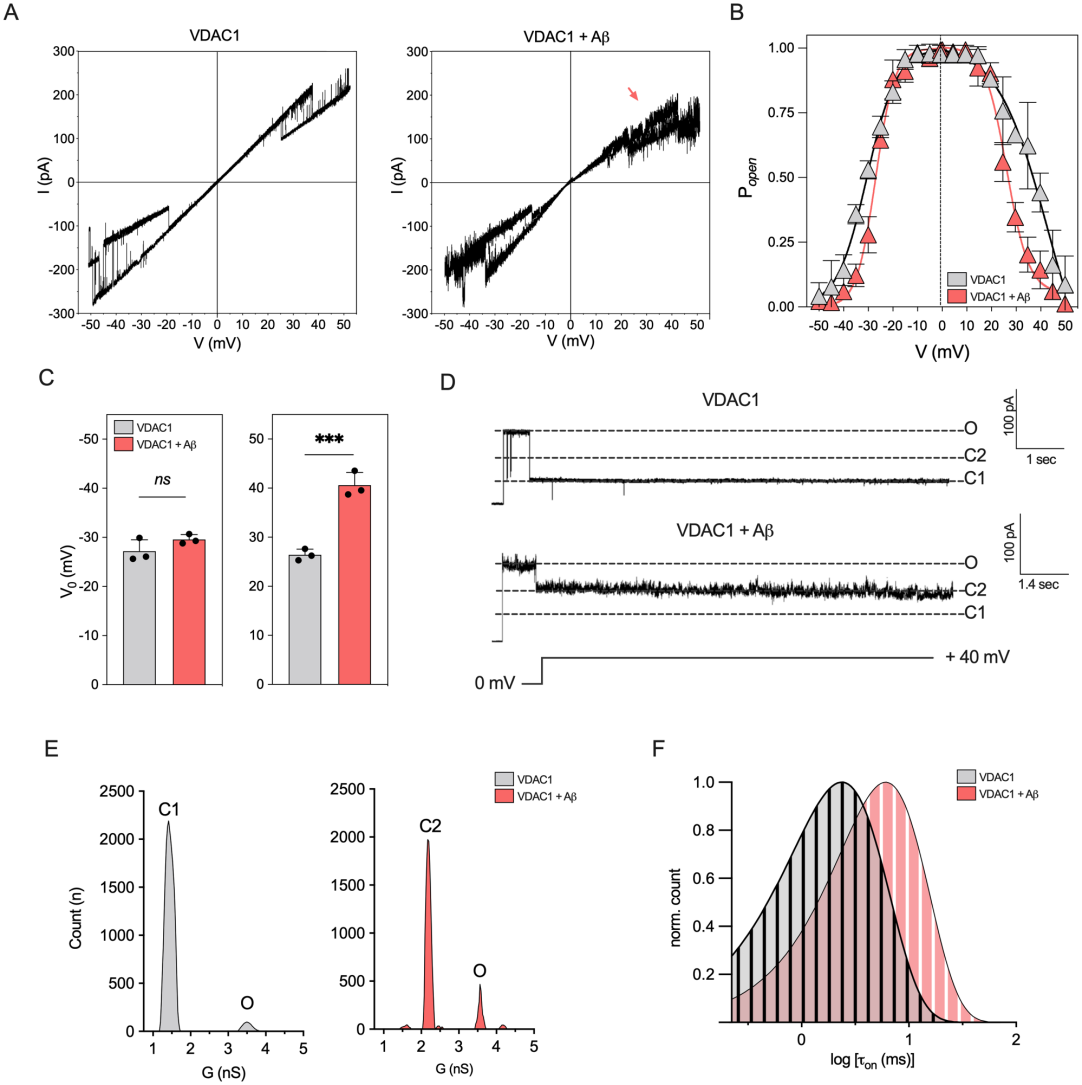
Voltage dependency and closure kinetics of VDAC1 are reduced upon the addition of Aβ oligomers. (A) Representative I/V plots, obtained by plotting the current as a function of clamp voltage, achieved for VDAC1 before (left plot) and after the addition of 0.3 μM Aβ oligomers (right plot). The plots derive from a triangular voltage ramp (±50 mV of amplitude) in symmetrical 1 M KCl buffer. The red arrow highlights the loss of voltage dependency in the presence of Aβ oligomers at positive potentials. An *n* = 3 independent experiments were performed. (B) The bell‐shaped plots of open probability (P_open_) showing the open state as a function of the voltage applied. An *n* = 3 independent experiments were performed. (C) The gating parameter voltage value (*V*_0_) associated with the transition of VDAC1 from open to closed state. Data were expressed as a mean ± SEM of *n* = 3 independent experiments and statistically analyzed by unpaired *t* test, with ***p < 0.001; ns, not significant.  (D) Representative current traces of single‐channel analysis of VDAC1 before (left trace) and after the addition of 0.3 μM Aβ oligomers (right trace). Traces were recorded at the constant voltage of +40 mV in symmetrical 1 M KCl buffer. Currents through the open (O) and two distinct closed states (C1 and C2) of the channel are indicated by dashed lines. An *n* = 3 independent experiments were performed. (E) Quantitative analysis of VDAC1 conductance as a function of the events relative to the traces displayed in (A). (F) Log‐binned distributions of the open time (*τ*_on_) obtained for VDAC1 before and after the addition of 0.3 μM Aβ oligomers recorded at the constant voltage of +40 mV. Solid lines are logarithmic single‐exponential fittings. An *n* = 3 independent measurements were performed.

To investigate this aspect, a triangular voltage wave in the range of amplitude of ±50 mV was applied to reconstituted VDAC1 before and after the addition of 0.3 μM Aβ oligomers (Figure S4A) and the derived current/voltage (I/V) plots were analyzed. As displayed in Figure 2A, the transition of VDAC1 from open to closed state started at ±20–30 mV, as expected, while the addition of Aβ oligomers prevented the achievement of stable closed states, reducing the conductance loss upon the application of high voltages. This effect was particularly evident at positive membrane potentials (Figure 2A, red arrow) and became most obvious in the analysis of the relative *G*/*G*_0_ conductance (Figure S4B) and the channel probability to be open (P_open_), both calculated as a function of the voltage. As displayed in Figure 2B, the P_open_ bell‐shaped curve, indicating the typical symmetrical voltage dependency at both positive and negative potentials, was less steep in the presence of Aβ oligomers, especially for positive voltages applied, showing remarkable differences in gating activity exclusively at potentials between +30 and +40 mV. Moreover, the voltage value associated with the transition of VDAC1 from open to closed state (*V*_0_) was significantly higher in the presence of Aβ oligomers at positive, but not negative, potentials (*p* = 0.001, Figure 2C).

Given these results, we investigated the behavior of VDAC1 upon the application of a constant potential of +40 mV. As reported in the higher trace of Figure 2D and in Figure 2E, VDAC1 underwent fast gating from a high‐ to low‐conducting state, reaching an average value of *G* = 1.5 ± 0.13 nS in the closed state (state C1). However, in the presence of Aβ oligomers, the channel transits toward a closed state characterized by a higher conductance (*G* = 2.25 ± 0.09 nS, state C2, Figure 2D,E).

Current traces were also exploited to determine how Aβ oligomers affect the closure kinetic of VDAC1 by calculating the dwell time in the open state (*τ*_on_), that is, the distribution of the time spent by the channel in the open state over the entire time period of the analysis. As displayed in Figure 2F, the *τ*_on_ followed an exponential distribution in the presence and absence of Aβ oligomers, although a rightward shift of the *τ*_on_ distribution was observed in the exclusive presence of Aβ oligomers.

These data confirm that Aβ oligomers partially hamper the achievement of the typical low‐conducting state of VDAC1 at high voltages.

### 2.3 Aβ Oligomers Profoundly Change the Ion Selectivity of VDAC1

**FIGURE 3:**
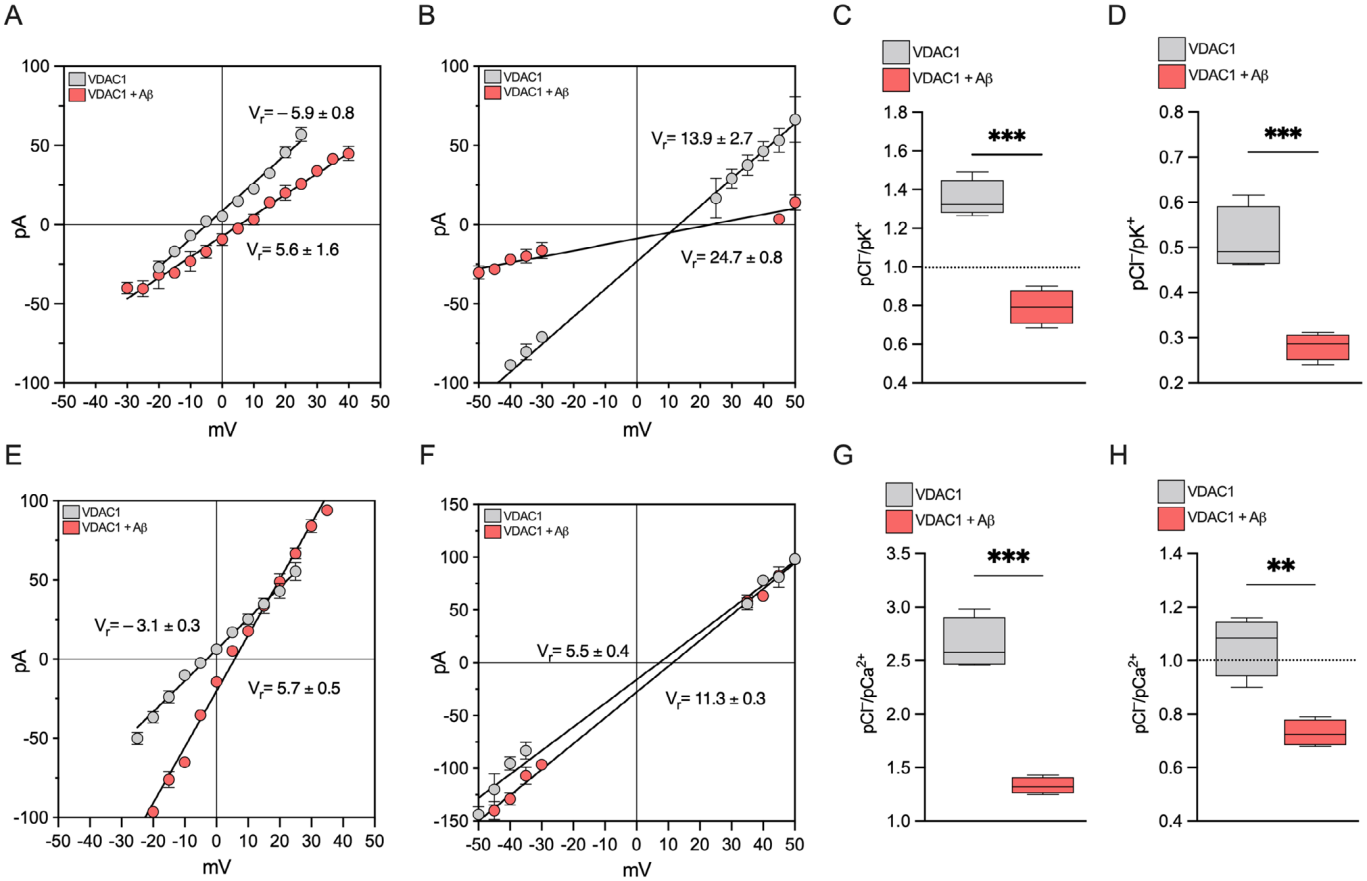
Aβ oligomers significantly alter the ion selectivity of VDAC1. (A, B) I/V plots of VDAC1 performed in a 10‐fold gradient of 0.1/1 M KCl for the selectivity analysis in the high‐conducting (A) or low‐conducting (B) state. Currents were obtained by the application of a triangular voltage wave (amplitude ±50 mV). The two different regression lines (solid lines) are for VDAC1 before (gray line) or after the addition of 0.3 μM Aβ oligomers (red line). The intercept lines with the I axis identify the Vr values. Data are expressed as a mean ± SEM of *n* = 4 independent experiments. (C, D) Permeability ratios (P_Cl_^−^/P_K_^+^) calculated from corresponding reversal potentials in the high‐conducting (C) or low‐conducting (D) state by the Goldman–Hodgkin–Katz equation for VDAC1 before and after the addition of 0.3 μM Aβ oligomers. (E, F) I/V plots of VDAC1 performed in a 10‐fold gradient of CaCl_2_ for the selectivity analysis in the high‐conducting (E) or low‐conducting (F) state. Currents were obtained by the application of a triangular voltage wave (amplitude ±50 mV). Regression lines are for VDAC1 before (gray line) or after the addition of 0.3 μM Aβ oligomers (red line). The intercept lines with the I axis identify the Vr values. Data are expressed as a mean ± SEM of *n* = 3 independent experiments. (G, H) Permeability ratios (P_Cl_^−^/P_Ca_^2+^) calculated from corresponding reversal potentials in the high‐conducting (G) or low‐conducting (H) state by the adapted Goldman–Hodgkin–Katz equation for VDAC1 before and after the addition of 0.3 μM Aβ oligomers. Data are expressed as a mean ± SEM of *n* = 4 independent experiments and statistically analyzed by unpaired *t* test, with **p < 0.01 and ***p < 0.001.

As for the conductance, the ion selectivity of VDAC1 varies in accordance with the potential applied, showing a slight preference for anion or cation in the open and closed states, respectively (Colombini 2016). To assess whether Aβ oligomers may alter this crucial parameter, the reversal potential (Vr), consisting of the voltage value corresponding to zero current in a 10‐fold gradient of KCl (1 M *cis* vs. 0.1 M *trans*), was measured upon application of a triangular voltage wave of ±50 mV and subsequently used to calculate the permeability ratio of Cl^−^ to K^+^ (P_Cl_^−^/P_K_^+^) in both high‐ and low‐conducting states by the Goldman–Hodgkin–Katz equation. Under these experimental conditions, VDAC1 displayed the typical slight anion selectivity in the open state with a negative Vr = −5.9 ± 0.8 mV (Figure 3A) and a moderate preference toward cations in the closed state characterized by a positive Vr = 13.9 ± 2.7 mV (Figure 3B). Surprisingly, the addition of Aβ oligomers to reconstituted VDAC1 led to a profound change in the channel selectivity, with the Vr being positive at both open and closed states (5.6 ± 1.6 mV and 24.7 ± 0.8 mV, respectively, Figure 3A,B). These data suggest that Aβ oligomers meaningfully change the permeability ratio P_Cl_^−^/P_K_^+^, conferring the porin a slight selectivity for cation already in the open state. In fact, the P_Cl_^−^/P_K_^+^ of VDAC1 in the open state was significantly reduced upon the addition of Aβ oligomers (1.35 ± 0.10 vs. 0.79 ± 0.09, *p* < 0.001, *n* = 4, Figure 3C). The pore preference toward positively charged ions is even more pronounced in the closed state, where the P_Cl_^−^/P_K_^+^ of VDAC1 varies from 0.51 ± 0.07 to 0.28 ± 0.03 upon Aβ oligomers addition (*p* = 0.001, *n* = 4, Figure 3D).

Being VDAC1 crucial for the maintenance of mitochondrial calcium homeostasis (De Stefani et al. 2011), we repeated the selectivity experiments in a 10‐fold gradient of CaCl_2_. Previous electrophysiological analyses suggest that the presence of small positive ions, such as Ca^2+^, does not affect the voltage gating or the porin functioning, as VDAC1 is poorly permeable to Ca^2+^, especially in the high‐conducting state (Tan and Colombini 2007). Consistent with the literature, our experimental conditions revealed a moderate preference for anions in the open state (Vr = −3.1 ± 0.3, Figure 3E) and a slight preference for cations in the closed state (Vr = 5.5 ± 0.4 mV, Figure 3F) for VDAC1 alone. Once again, the addition of Aβ oligomers on reconstituted VDAC1 promoted a significant changes in the selectivity, in line with what was observed in the previous experiments, both Vr values being increased (5.7 ± 0.5 and 11.3 ± 0.3 in the open and closed states, respectively, Figure 3E,F). Most interestingly, Aβ oligomers increased the VDAC1 permeability for Ca^2+^: in fact, the P_Cl_^−^/P_Ca_^2+^ of VDAC1 varies from 2.46 ± 0.18 to 1.33 ± 0.07 in the presence of Aβ oligomers (*p* < 0.001, *n* = 4, Figure 3G); a similar, but less pronounced, effect was observed in the closed state where the P_Cl_^−^/P_Ca_^2+^ of VDAC1 varies from 1.05 ± 0.1 to 0.73 ± 0.05 upon Aβ oligomers addition (*p* = 0.0017, *n* = 4, Figure 3H).

Altogether, these data indicate that, in the presence of Aβ oligomers, VDAC1 decreases the selectivity for anions typical of the open state, increasing its affinity for cations and, in particular, for calcium.

### 2.4 Aβ Oligomers Impair Mitochondrial Respiration and Cell Viability in the Neuroblastoma Cell Line

To investigate the effect of Aβ oligomers on mitochondrial functions in a more physiological environment, we analyzed the respiratory profile of neuroblastoma SH‐SY5Y cells by high‐resolution respirometry (HRR) after Aβ exposure. To increase the efficiency of oligomer internalization, we used digitonin to obtain a transient and mild permeabilization of the plasma membranes. The amount of detergent was previously determined by titration as the minimum concentration of digitonin able to abolish the oxygen consumption in the shorter timeframe of exposure due to the loss of the endogenous energetic substrates through permeabilized membranes (Figure S5A). Notably, the treatment with digitonin did not affect the mitochondrial membranes integrity, as the succinate‐stimulated respiration did not vary upon the addition of cytochrome c (CytC; Figure S5B), nor did it permanently damage the plasma membranes, as demonstrated by the complete recovery of respiration after 60 min following the detergent treatment (Figure S5C).

**FIGURE 4:**
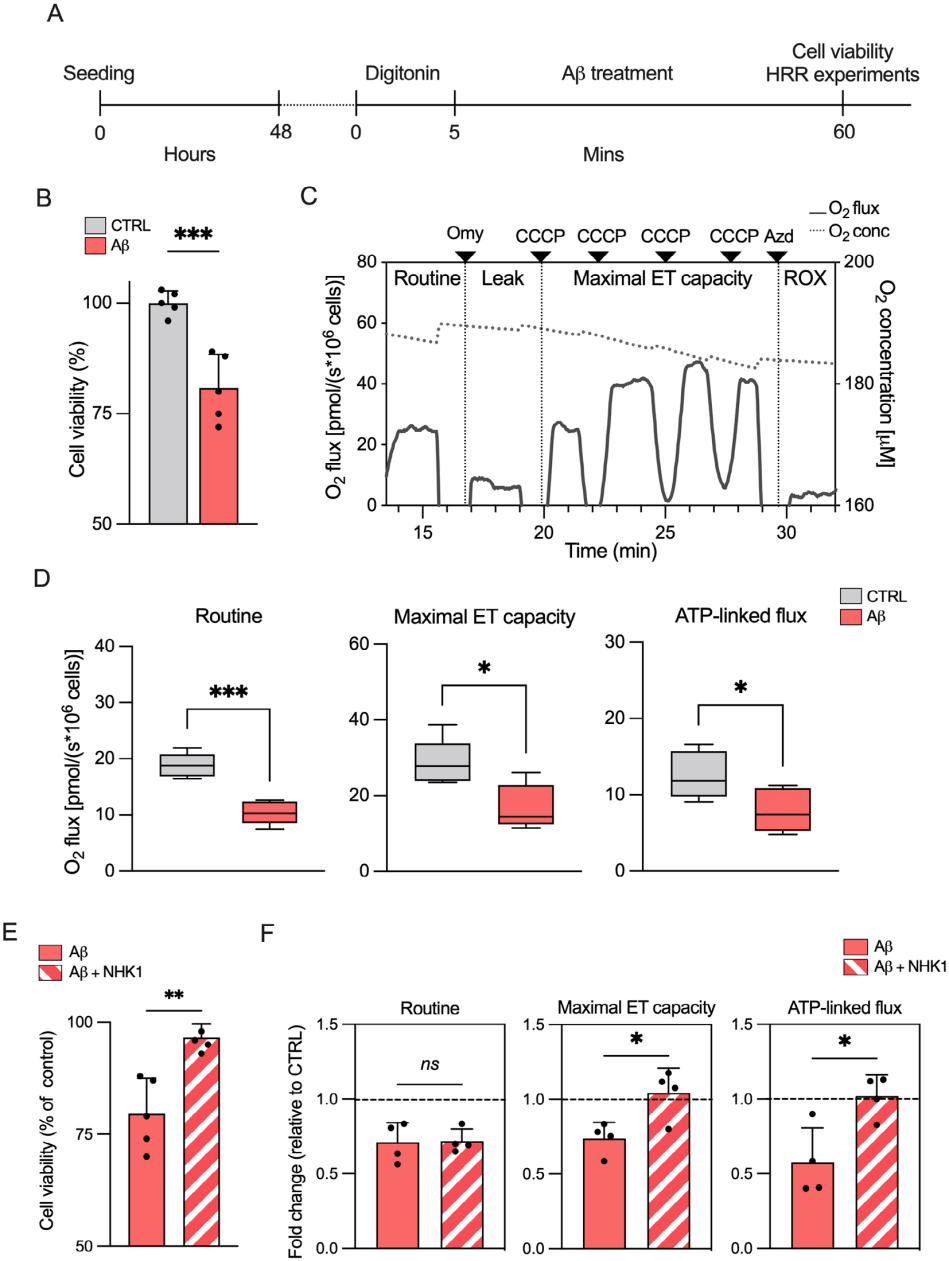
Aβ oligomers impair the respiratory profile of SH‐SY5Y cells. (A) Timeline representing the experimental approach used here for SH‐SY5Y cells. After seeding, cells were treated with 30 μg/mL digitonin for 5 min to transiently permeabilize the plasma membranes, and incubated with Aβ oligomers at a final concentration of 1 μM for an additional 60 min. Then, cells were immediately used for viability or HRR experiments. (B) Analysis of cell viability of SH‐SY5Y cells treated or not with Aβ oligomers performed by Trypan blue exclusion assay. Data are expressed as a percentage of the untreated control and as a mean ± SEM of *n* = 5 independent experiments. Data were statistically analyzed by unpaired *t* test, with ***p < 0.001. (C) Representative curve of the mitochondrial respiratory profile of untreated SH‐SY5Y cells assayed by HRR. The respiratory states Routine, Leak, maximal ET capacity, and ROX were analyzed after the addition of specific substrates, uncouplers, and inhibitors. Omy, oligomycin; CCCP, carbonyl cyanide 3‐chlorophenylhydrazone; Azd, sodium azide. (D) Quantitative analysis of the oxygen consumption rates relative to Routine, maximal ET capacity, and ATP‐linked flux of SH‐SY5Y treated or not with Aβ oligomers. Data are expressed as pmol/s per million cells and as a median ± SEM of *n* = 5 independent experiments. Data were statistically analyzed by unpaired *t* test, with *p < 0.05 and ***p < 0.001. (E) Analysis of cell viability by Trypan blue exclusion assay performed in transiently permeabilized SH‐SY5Y cells exposed to 1 μM Aβ oligomers or co‐incubated with NHK1 peptide at a final concentration of 10 μg/mL. Data are expressed as the percentage of untreated cells (control) and as a mean ± SEM of *n* = 4 independent experiments. Data were statistically analyzed by unpaired *t* test, with **p < 0.01. (F) Quantitative analysis of the oxygen consumption rates relative to Routine, maximal ET capacity, and ATP‐linked flux by HRR of permeabilized SH‐SY5Y cells treated as in (A). Data are expressed as fold change of untreated cells (control) and as a mean ± SEM of *n* = 4 independent experiments. Data were statistically analyzed by unpaired *t* test, with *p < 0.05; ns, not significant.

Confluent SH‐SY5Y cells were treated with digitonin at the final concentration of 30 μg/mL for 5 min, after which the solution was removed and the cells were exposed or not to 1 μM of Aβ oligomers for an additional hour (Figure 4A). To assess the effectiveness of the protocol, cell viability was analyzed by the Trypan blue exclusion assay. As reported in Figure 4B, the exposure of permeabilized cells to the presence of Aβ oligomers promoted a significant reduction of viability of ~20% in comparison to control cells, that is, permeabilized cells not exposed to Aβ oligomers (*p* = 0.0005, *n* = 5). Then, the same approach was used to determine the respiratory profile of neuroblastoma cells by applying a substrate‐uncoupler‐inhibitor titration (SUIT) protocol displayed in Figure 4C along with a representative curve relative to control cells. The comparative analysis in Figure 4D indicates that Aβ oligomers significantly affect the respiration in all the respiratory states here assayed. Precisely, Aβ oligomers reduced the oxygen flow supported by endogenous substrates, the so‐called Routine state, by about 45% (*p* = 0.0002, *n* = 5), and the maximal capacity of the electron transport (ET) chain, measured upon titration with the uncoupler carbonyl cyanide 3‐chlorophenylhydrazone (CCCP), by about 40% (*p* = 0.015, *n* = 5). Most interestingly, the oxygen flux devoted to ADP phosphorylation, here calculated as a function of the dyscoupled respiration (Leak) achieved by blocking the ATP synthase with oligomycin, was reduced by about 37% (*p* = 0.039, *n* = 5).

These data indicate that the entry of Aβ oligomers within neuroblastoma cells dramatically affects mitochondrial functionality, reducing respiration.

### 2.5 A VDAC1‐Targeted NHK1 Peptide Counteracts Aβ Oligomers Harmful Effects on Mitochondria

NHK1 is a small synthetic, cell‐penetrating peptide that corresponds to the first 11 amino acids of the N‐terminal domain of human HK1, the most common physiological interactor of VDAC1. As previously demonstrated by our group, NHK1 peptide binds VDAC1 and reduces the interaction of mitochondrial porin with misfolded proteins in neurodegenerative contexts (Magrì et al. 2016, 2021). Thus, considering the characteristics of NHK1, the peptide was here used to test whether the mitochondrial toxicity linked to Aβ oligomers depends, at least in part, on direct interaction with VDAC1. To this end, permeabilized SH‐SY5Y cells were exposed to NHK1 at the final concentration of 10 μg/mL. As shown in Figure S6, NHK1 itself did not exert any harmful effect in this model, the cell viability and the respiratory profile being unaffected by the presence of the peptide. Then, the experimental approach previously illustrated in Figure 4A was applied to compare viability and respiration in cells exposed to Aβ oligomers, or to a combination of both Aβ oligomers and NHK1. As displayed in Figure 4E, the presence of NHK1 significantly mitigated the toxicity of Aβ oligomers, increasing the cell viability by about 18% in comparison to cells exposed exclusively to Aβ oligomers (*p* = 0.0020, *n* = 4). Concurrently, NHK1 fully rescued the oxygen consumption related to maximal ET capacity and to ATP production (*p* = 0.021 and *p* = 0.017, respectively, vs. cells exposed to the Aβ oligomers alone, *n* = 4, Figure 4F).

In summary, these data strongly support the hypothesis that VDAC1 represents the primary driver of mitochondrial dysfunction linked to Aβ oligomers in our experimental model.

### 2.6 NHK1 Peptide Protects Primary Cortical Neurons From Aβ Oligomers Toxicity

**FIGURE 5:**
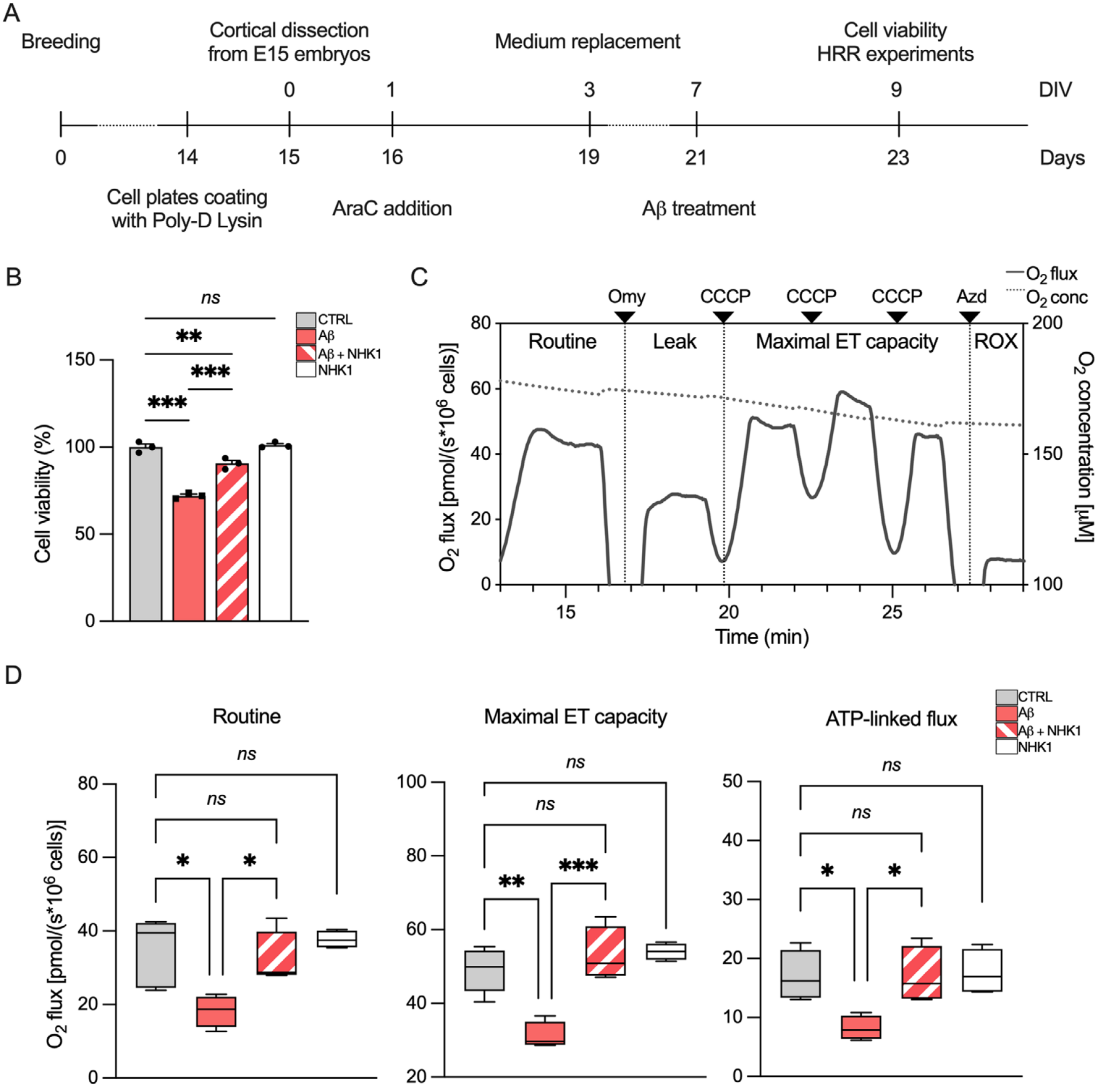
NHK1 peptide counteracts Aβ oligomers toxicity in primary cortical neurons. (A) Timeline representing the experimental approach here used for primary cortical neurons preparation and treatment. Primary cultures were prepared from rats at embryonic day 15. Mechanically dissociated cortical neurons were seeded, treated with Ara‐C, and kept for 3 days before medium replacement. Cell viability and HRR experiments were then performed on day in vitro (DIV) 9. (B) Analysis of cell viability by MTT assay of primary cortical neurons exposed or not to 2 μM Aβ oligomers and/or 10 μg/mL NHK1 peptide for 48 h. Values are expressed as percentage of the untreated control and as a mean ± SEM of *n* = 3 independent experiments each performed at least in triplicate. Data were statistically analyzed by one‐way ANOVA followed by Tukey test, with **p < 0.01 and ***p < 0.001; ns, not significant. (C) Representative curve of mitochondrial respiratory profile of untreated primary cortical neurons by HRR. The respiratory states Routine, Leak, maximal ET capacity, and ROX were analyzed after the addition of specific substrates, uncouplers, and inhibitors. Omy, oligomycin; CCCP, carbonyl cyanide 3‐chlorophenylhydrazone; Azd, sodium azide. (D) Quantitative analysis of the oxygen consumption rates relative to Routine, maximal ET capacity, and the ATP‐linked flux of primary cortical neurons exposed or not to Aβ oligomers and/or NHK1 peptide. Data are expressed as pmol/s per million cells and as a median ± SEM of *n* = 4 independent experiments. Data were statistically analyzed by one‐way ANOVA followed by Tukey test, with *p < 0.05, **p < 0.01 and ***p < 0.001; ns, not significant.

The previous data highlighted the potential of NHK1 as a pharmacological tool for counteracting mitochondrial toxicity induced by Aβ oligomers. To test this hypothesis, we moved toward a more relevant neuronal model, the rat‐derived primary cortical neurons. To obtain pure mature cultures, neurons were prepared from rat embryos at day 15 and treated with 2 μM of Aβ oligomers after 7 days in vitro (Figure 5A). As expected, the 48 h treatment with Aβ oligomers promoted a reduction of cell viability of about 30% in comparison with the untreated control (*p* < 0.001, *n* = 3, Figure 5B). Interestingly, the addition of NHK1 3 h before the Aβ treatment was able to prevent Aβ toxicity by counteracting cell death (*p* < 0.001, *n* = 3, Figure 5B). Notably, no significant effects were detected after the addition of NHK1 to untreated neurons (Figure 5B).

The impact of Aβ oligomers and NHK1 on the mitochondrial respiratory profile of primary cortical neurons was then analyzed by applying the SUIT protocol shown in Figure 5C along with a representative curve relative to untreated primary cortical neurons. As reported in Figure 5D, the administration of Aβ oligomers resulted in a significant reduction in oxygen consumption in all the states. Precisely, we noticed a reduction of about 47% in the Routine (*p* = 0.014, *n* = 4), 36% in the maximal ET capacity (*p* = 0.0011, *n* = 4), and 52% in the flux linked to the ADP phosphorylation (*p* = 0.021, *n* = 4) in comparison to the cells exclusively exposed to Aβ oligomers. Strikingly, the addition of NHK1 was observed to fully restore the oxygen consumption of Routine (*p* = 0.0485, *n* = 4); maximal ET capacity (*p* = 0.0003, *n* = 4); and ATP‐linked flux (*p* = 0.0315, *n* = 4, Figure 5D) in comparison to Aβ oligomers‐treated cells, thereby reaching values comparable to those of the control. As observed for SH‐SY5Y, the administration of NHK1 alone to control cells did not result in any toxic effects (Figure 5D).

In conclusion, the overall data presented provide evidence that NHK1 peptide may serve as a potential pharmacological agent for counteracting the mitochondrial dysfunction in AD.

## 3 Discussion

The maintenance of neuronal and synaptic functions in aging and pathological conditions strictly depends on the efficiency of the continuous exchange of energetic substrates between the mitochondria and the rest of the cell. This process is ensured by the presence of VDAC1 on the OMM for at least three different reasons. First, VDAC1 is the most abundant integral protein embedded in the OMM with a β‐barrel structure, exceeding the second and third most representative proteins by one and two orders of magnitude, respectively, a feature that confers to the membrane itself the typical sieve‐like aspect (Ellenrieder et al. 2019; Gonçalves et al. 2007, 2008). Second, VDAC1 is not merely a channel: rather, it functions as a molecular switch in the regulation of the overall mitochondrial bioenergetics. As evinced from the literature, the genetic inactivation of the VDAC1 gene results in a complete rewiring of the mitochondrial metabolism that, in human cells, is only partially mitigated by the presence of the other VDAC isoforms (Magrì et al. 2020, 2023). Moreover, the expression of the VDAC1 gene is finely regulated in specific diseases or under stress conditions, such as nutrient deprivation and hypoxia (Pappalardo et al. 2023; Zinghirino et al. 2021; Guarino et al. 2020), and was recently used to mitigate mitochondrial dysfunction in pathological contexts (Magrì et al. 2024). Third, VDAC1 is the physiological receptor of cytosolic enzymes, but also of misfolded proteins in several neurodegenerative diseases (Magri and Messina 2017). For these reasons, VDAC1 has rapidly emerged as a putative pharmacological target in the treatment of various pathologies, including AD in which the mitochondrial dysfunction is partially attributable to the toxic interaction of intracellular Aβ oligomers with the porin.

With the aim to expand our knowledge on this specific aspect, we investigated the impact on the electrophysiological features of VDAC1 of Aβ oligomers, here prepared according to a precise procedure to ensure the formation of toxic species. Specifically, we started from a batch of commercially available HFIP‐treated Aβ_1–42_, which was further lyophilized and dissolved in dimethyl sulfoxide (DMSO) to remove any preexisting β‐sheet secondary structure or seeds. Oligomers were then prepared from the monomeric starting solution by using the well‐established Lambert protocol and characterized by Western blot analysis to confirm different patterns of band distribution, which are known to underline different activities on cells. Indeed, as a control, we tested the biological activity of the oligomers preparation on primary cells, observing a reduction of cell viability consistent with the previous literature. Furthermore, PLB experiments corroborated the previous observation that oligomeric forms of Aβ enhance the average conductance of the mitochondrial porin (Smilansky et al. 2015). It should be noted that neither the use of a scrambled peptide, whose aggregates might interact with cellular components and/or proteins in unpredictable ways, nor the monomeric Aβ, which has its own biological activity, cannot be considered a reliable control.

Our model demonstrated that Aβ_1–42_ oligomers alter the conductance of VDAC1 primarily by disturbing the open state at low voltages, thereby facilitating the attainment of an unstable state characterized by a higher conductance. This, in turn, affects the voltage dependency, as well as the kinetic process regulating the transition from high‐ to low‐conducting states at potential values which typically induce a stable closure of VDAC1. Notably, a reduction or loss of the voltage dependency has been already observed for VDAC1 in the presence of interacting molecules, such as the copolymer Koenig polyanion (Colombini et al. 1987; Benz et al. 1988), and upon selective mutagenesis of specific amino acid residues or whole domains (Runke et al. 2006; Reina et al. 2010, 2013). In any case, these findings suggest that Aβ oligomers have the potential to bind and stabilize VDAC1 in an unphysiological conformation.

Nonetheless, the most striking outcome is that Aβ oligomers have been shown to reduce the typical anion selectivity of VDAC1 in the open state, simultaneously favoring the appearance of a cationic selectivity. These changes may alter the import and efflux mechanisms involving many important ions (such as Ca^2+^) and charged molecules across VDAC1, a function of crucial importance for the maintenance of mitochondrial homeostasis (Rostovtseva and Colombini 1997). Since selectivity is a direct consequence of the net charge facing the channel lumen, it can be speculated that, once again, Aβ oligomers may modify the three‐dimensional structure or mask specific VDAC1 residues, in any case altering the charge distribution of the protein. Anyway, this is not the first case of a high cationic state observed in VDAC proteins: a similar feature has been previously noticed in yeast VDAC2, despite the high degree of similarity with the human VDAC1 in terms of primary sequence and three‐dimensional structure (Guardiani et al. 2018; Magrì et al. 2019; Di Rosa et al. 2021).

In line with these considerations, in our opinion, it is particularly relevant that Aβ oligomers increase the affinity of VDAC1 for Ca^2+^, as confirmed by selectivity experiments performed in a CaCl_2_ gradient. Indeed, mitochondrial Ca^2+^ signaling has been implicated in the regulation of apoptosis, as the rise of mitochondrial Ca^2+^ concentration is prodromal to the organelle swelling and the release of pro‐apoptotic factors from mitochondria (Pinton et al. 2001). In this specific context, VDAC1 mediates the efficient transfer of Ca^2+^ from the endoplasmic reticulum (ER) to the mitochondria, being associated with the inositol 1,4,5‐trisphosphate receptor and the glucose‐regulated protein 75 (Grp75) at the ER‐mitochondrial contact sites (MERCS) (De Stefani et al. 2011; Rosencrans et al. 2021). In this regard, Grp75 is also known to interact with the VDAC3 isoform (Messina et al. 2014). Therefore, we can speculate that the accumulation of Aβ oligomers in neurons may also alter the structure and/or function of MERCS, thereby promoting a dysregulation of calcium homeostasis and cell death.

The changes in selectivity and the alteration of the main VDAC1 electrophysiological features correlate with an overall reduction of the mitochondrial respiratory rates here observed. Indeed, while oxygen consumption is ultimately dependent on the functioning of the ET chain, it is also affected by the availability of substrates within the mitochondria. Not coincidentally, the knockout of the VDAC1 gene is sufficient to compromise the respiratory profile of human cells (Magrì et al. 2023). Moreover, this finding is strengthened by the overlapping results exhibited by the two cellular models employed in this study. In particular, the use of permeabilized SH‐SY5Y cells enabled the assessment of damage caused by the direct interaction of Aβ oligomers with the OMM, which was achieved within a brief exposure time. Notably, this approach avoided any additional side effect that might otherwise be caused by Aβ accumulation. In contrast, the exposure of intact primary cortical neurons to Aβ oligomers for 48 h allowed the evaluation of mitochondrial dysfunction upon a gradual accumulation of Aβ within the cells. In both cases, the presence of Aβ oligomers induces a significant reduction of cell viability and oxygen consumption in all the states here analyzed, with a particularly pronounced impact on the ATP‐related flux, in accordance with previous observations (Sotolongo et al. 2020; Karapetyan et al. 2022).

Interestingly, the toxicity of Aβ oligomers was strikingly attenuated by the co‐incubation with NHK1 peptide, a synthetic mitochondrial‐targeted molecule that mimics the N‐terminal domain of HK1. Consistent with previous observations, this domain is indeed critical in mediating HK1 binding to VDAC1 (Abu‐Hamad et al. 2008). NHK1 was previously designed and patented by our group with the specific purpose of interfering with the interaction of misfolded proteins with VDAC1 and preventing their deposition on the cytosolic surface of mitochondria (Messina et al. 2020a, 2020b). This process significantly contributes to the pathogenesis of several familial forms of amyotrophic lateral sclerosis (ALS) linked to mutations in the gene encoding the antioxidant enzyme Cu/Zn superoxide dismutase (SOD1) (Israelson et al. 2010). The ability of NHK1 to bind and modulate VDAC1 in a similar manner to the endogenous protein, as well as its capacity to reduce the formation of toxic VDAC1‐SOD1 mutant aggregates in ALS, has already been demonstrated in both cell‐free assays and purified mitochondria from motor neuronal‐like cells (Magrì et al. 2016). Furthermore, being able to spontaneously cross biological membranes and reach mitochondria, NHK1 exerted a beneficial effect on the mitochondrial functionality of the same cell line. In particular, NHK1 ameliorated the overall respiratory profile of the ALS model and, similarly to what was observed here, it completely restored the respiration linked to ADP phosphorylation (Magrì et al. 2021). Therefore, based on the above and considering that ALS and AD, as well as other neurodegenerative diseases, share common mechanisms for the accumulation of misfolded molecules at the mitochondrial level, we can hypothesize that, by binding VDAC1, NHK1 competes with Aβ oligomers for the same binding site, thus preventing mitochondrial dysfunction. It is, however, not the first time that VDAC1 has been used as a pharmacological target in AD. In a recent study, a VDAC1‐interacting molecule, VBIT‐4, was successfully employed to mitigate neuronal cell death and inflammation that depend on Aβ‐induced overexpression of VDAC1 in a transgenic mice model of AD (Verma et al. 2022).

We are aware that our study leaves some open questions about important aspects of the interaction between the molecules here studied, such as the exact domain(s) of VDAC1 and the specific amino acid residues of Aβ and NHK1 directly involved in the interaction, although some hypotheses have already been proposed (Smilansky et al. 2015; Magrì et al. 2021). Also, very little is known about the involvement of the other VDAC isoforms in the pathological context of AD. While VDAC3 is generally considered a sensor of oxidative stress (Reina et al. 2022), VDAC2 plays an important role in the regulation of apoptosis and mitochondrial calcium signaling, as it has been found to be associated with ryanodine receptor 2 for Ca^2+^ transfer at the level of the sarcoplasmic reticulum–mitochondrial junction in the heart (Min et al. 2012).

In any case, the results presented here establish a reasonable link between mitochondrial dysfunction in AD and the VDAC1‐Aβ oligomers interaction, bringing new elements to the complex puzzle represented by this field of investigation. More interestingly, our results show the validity of counteracting mitochondrial dysfunction in AD by using VDAC1‐targeted molecules. In particular, the beneficial effect produced by NHK1 peptide makes it clear how promising this approach is for the treatment of AD and other diseases in which VDAC1 is crucially involved (Shteinfer‐Kuzmine et al. 2019; Risiglione et al. 2021; Hoogerheide et al. 2017; Conti Nibali et al. 2024).

## 4 Methods

### 4.1 Chemicals

All chemicals used in this work are of the highest grade of purity and were purchased from Sigma‐Aldrich (St. Louis, MO, USA) unless otherwise specified.

### 4.2 Expression and Purification of Recombinant Human VDAC1

Human, recombinant 6xHis‐tagged VDAC1 was produced by heterologous expression in BL21 (DE3) *E. coli* strain and extracted from inclusion bodies, as recently detailed (Conti Nibali et al. 2025). Briefly, isolated inclusion bodies were resuspended in lysis buffer (50 mM Tris–HCl, 2 mM EDTA, 20% sucrose, pH 8.0). After sonication and centrifugation, inclusion bodies were resuspended in Wash buffer (20 mM Tris–HCl, 300 mM NaCl, 2 mM CaCl_2_, pH 8.0) and solubilized in equilibration buffer A (20 mM Tris–HCl, 300 mM NaCl, 8 M Urea, pH 8.0) for 3 h at 4°C under constant stirring. Recombinant VDAC1 was purified by affinity chromatography with the HisTrap HF 5 mL affinity column (Cytiva). The column was washed with five column volumes (CV) of equilibration buffer A containing 30 mM imidazole and the protein was eluted with eight CV of equilibration buffer A containing 150 mM imidazole.

### 4.3 In Vitro Refolding and Size‐Exclusion Chromatography

VDAC1 refolding was performed by dropwise dilution (100 μL/min) of protein solution into Refolding buffer (25 mM Tris–HCl, 300 mM NaCl, 1 mM EDTA, 5 mM DDT, 1% [w/v] LDAO, pH 8.0) at a pellet:buffer ratio of 1:20 (w/v). The procedure was carried out at 4°C for 3 h. The refolded protein was then dialyzed overnight at 4°C against a dialysis buffer (25 mM Tris–HCl, 300 mM NaCl, pH 8.0). To reduce the detergent concentration, the protein was applied to a HisTrap HP 5 mL affinity column, washed with five CV of equilibration buffer B (25 mM Tris–HCl, 300 mM NaCl, 0.1% (w/v) LDAO, pH 8.0) containing 30 mM imidazole, and eluted with eight CV of equilibration buffer B containing 250 mM imidazole. Refolded protein was concentrated by using Amicon Ultra‐30KDa (Millipore, Burlington, MA, USA). Finally, the protein solution was applied to a Superdex 200 increase 10/300 column (Cytiva), and the monomeric form was isolated by size‐exclusion chromatography by elution with SEC buffer (20 mM Tris–HCl, 50 mM NaCl, 0.1% [w/v] LDAO, pH 8.0). The monomeric state of the target protein was verified by SDS‐PAGE.

### 4.4 Peptides

Aβ_1–42_ peptide was purchased from Bachem (Bubendorf, Switzerland, Cat. no. 4090148, purity ≥ 95%). To avoid the presence of any preformed aggregates, Aβ_1–42_ was initially dissolved in hexafluoro‐isopropanol at a concentration of 1 mg/mL and then lyophilized overnight. The lyophilized powder was dissolved in DMSO to obtain a stock solution with a final concentration of 5 mM. Aβ_1–42_ oligomers were prepared by diluting the stock solution in ice‐cold DMEM F‐12 w/o phenol red (Gibco, Thermo Fisher Scientific, Waltham, MA, USA) to a final concentration of 100 μM and allowed to oligomerize at 4°C according to the Lambert protocol (Lambert et al. 1998).

NHK1 peptide, corresponding to the 2–12 amino acid sequence of human HK1 (IAAQLLAYYFT), was synthesized and purchased from Proteogenix (Schiltigheim, France, purity ≥ 95%). NHK1 was dissolved in 50% ammonium hydroxide and used at a final concentration of 10 μg/mL or stored at 4°C for up to a week.

### 4.5 Western Blot Analysis

Characterization of Aβ oligomers and freshly solubilized monomers was carried out by Western blot. A volume of 15 μL of unheated samples was loaded onto a precast Bis‐Tris gel (Bolt 4%–12%, Life Technologies) with 2‐morpholin‐4‐yl ethanesulfonic acid. Proteins were transferred onto a nitrocellulose membrane (0.2 mm, Hybond ECL, Amersham Italia) by using a wet transfer unit Mini Blot Module (Life Technologies). Membranes were blocked in Odyssey blocking buffer (Li‐COR Biosciences) and incubated at 4°C overnight with anti‐Aβ N‐terminal 1–16 mouse monoclonal antibody 6E10 (1:800) (BioLegend). A secondary goat anti‐mouse antibody labeled with IR dye (1:20,000) was used at RT for 45 min. Hybridization signals were detected with the Odyssey CLx infrared imaging system (LI‐COR Biosciences, Lincoln, NE).

### 4.6 Analysis of VDAC1 Conductance

The refolded VDAC1 was reconstituted into a PLB system as previously described (Conti Nibali et al. 2021). An artificial bilayer made of asolectin from a soybean phospholipid mixture at a concentration of 20 mg/mL in *n*‐decane was formed on an aperture of 200 μM in a Derlin cuvette (Warner Instruments, Hamden, CT, USA). Membrane capacitances of 90–120 pF were accepted for proper lipid bilayers. Channel insertion was obtained by addition of ~40 ng of refolded and monomeric protein to the *cis* side of the cuvette containing 3 mL of KCl solution (1 M KCl, 10 mM Hepes, pH 7.0). Data were acquired using a Bilayer Clamp amplifier (Warner Instruments) at 100 μs/point, filtered at 300 Hz, and analyzed using the pClamp software (version 10, Molecular Devices, San Jose, CA, USA). Pore conductance (*G*) was calculated from current (I) measurements in the presence of an applied constant voltage (V) of +10 or +40 mV as the I/V ratio. The frequency distribution was calculated by normalizing the conductance values for the number of events. The impact of Aβ oligomers on the electrophysiological property of VDAC1 was investigated by adding 0.3 μM to both sides of the membrane. At least *n* = 4 independent reconstitution experiments were performed.

### 4.7 Analysis of Voltage Dependency

VDAC1 voltage dependency was measured in a symmetrical KCl solution by applying 10 MHz triangular voltage waves of ±50 mV for 100 s. Plots of the average conductance of VDAC1 as a function of voltage were obtained by the triangular voltage wave as recently reported (Foti et al. 2023). The relative conductance was calculated as *G*/*G*_0_, where *G* denotes average conductance at a given voltage and *G*_0_ denotes average conductance values calculated in the presence of the lowest applied potential. P_open_ analysis and V_0_ were obtained from single‐channel recording experiments by the application of a voltage range of ±50 mV with discrete steps of ±10 mV as previously performed in Queralt‐Martín et al. (2019). V_0_ was obtained from the fitting of the P_open_ versus V plot with the Boltzmann equation. The dwell‐time analysis for the VDAC1 open state was obtained from single‐channel recording experiments by the application of a constant voltage of +40 mV. The dwell‐time histogram was obtained by fitting the exponential log‐probability to the number of normalized events. At least *n* = 3 independent experiments for each analysis were performed.

### 4.8 Ion Selectivity Measurement

Ion selectivity was measured in 0.1 M/1 M *cis*/*trans* gradient of KCl or CaCl_2_. Permeability ratios P_Cl_^−^/P_K_^+^ were calculated from the reversal potential (Vr) using the Goldman–Hodgkin–Katz equation as reported in Conti Nibali et al. (2021). Evaluation of the permeability ratio P_Cl_^−^/P_Ca_^2+^ was performed as described in Alvarez and Latorre (2017) and Cubisino et al. (2024). Channel insertion was initially achieved in symmetrical 1 M KCl or CaCl_2_. After the insertion of at least one channel, the solution in cis was changed by perfusing ~10 chamber volumes, and a 10 MHz triangular voltage wave (±50 mV, 100 s) was applied. At least *n* = 4 independent experiments were performed.

### 4.9 Cell Cultures

The neuroblastoma cell line SH‐SY5Y was maintained in DMEM‐F12 (Gibco, Thermo Fisher Scientific) in a 1:1 proportion supplemented with 10% heat‐inactivated fetal bovine serum, 1% penicillin/streptomycin, and 2 mM l‐glutamine at 37°C, 5% CO_2_. Cultures of pure cortical neurons were obtained from rats at embryonic day 15 as previously described (Giuffrida et al. 2009). Cortical cells were dissected, mechanically dissociated, and seeded on 35 mm dishes, 24‐ or 96‐well plates pre‐coated with 0.1 mg/mL poly‐d‐lysine in Neurobasal Plus Medium (Gibco, Thermo Fisher Scientific) complemented with B27 Plus (Gibco, Thermo Fisher Scientific). Cells were incubated at 37°C with 5% CO_2_ in a humidified atmosphere. Cytosine arabinoside (1‐β‐d‐arabinofuranosylcytosine, Ara‐C) (3–10 μM) was added to the cultures 18 h after plating to avoid the proliferation of non‐neuronal elements and was kept for 3 days before medium replacement. Experiments have been performed on DIV 7–9. Animal care and experimentation were conducted in compliance with institutional guidelines.

### 4.10 Cell Viability Assays

Cell viability upon different treatments and conditions was assayed by Trypan blue exclusion assay or 3‐(4,5‐Dimethylthiazol‐2‐il)‐2,5‐difeniltetrazolium bromide (MTT), as previously detailed (Kanat and Selmanoğlu 2020). Trypan blue dye was added to the cell culture to exclude positive cells by counting. Primary cultures were incubated with 0.5 mg/mL of MTT for 2 h at 37°C and then lysed with DMSO. The formazan production was determined by measuring the absorbance at 570 nm using the 96‐well microplate reader Victor Nivo (PerkinElmer, Waltham, MA, USA). At least *n* = 4 independent experiments for each analysis were performed.

### 4.11 High‐Resolution Respirometry

The mitochondrial respiration of SH‐SY5Y and primary cortical neurons was assayed by HRR using the two‐chamber system O2k‐FluoRespirometer (Oroboros Instruments, Innsbruck, Austria). The SUIT protocol was adapted from previous reports (Risiglione et al. 2020; Leggio et al. 2022). First, the physiological oxygen consumption, corresponding to Routine state, was measured. Then, the dissipative flux (Leak state) was achieved by using oligomycin (Omy) at the final concentration of 1 μM for SH‐SY5Y and 5 μM for primary cultures. The maximal capacity of the ET chain was achieved by titration with 0.5 μM of the uncoupler CCCP. Finally, antimycin A (5 μM) was added to inhibit the ET chain enzymes and determine the residual oxygen consumption (ROX). The rate of oxygen consumption in all the respiratory states Routine, Leak, and maximal ET capacity was corrected for the ROX and expressed as pmol/s per million cells. The ATP‐linked flux was determined as the difference between Routine and Leak. Instrumental and chemical background fluxes were calibrated as a function of oxygen concentration using DatLab software (version 7.4, Oroboros Instruments). All the experiments were performed at 37°C in respirometric buffer Mir05 (Oroboros Instruments), under constant stirring (750 rpm). At least *n* = 4 independent experiments for each analysis were performed.

### 4.12 Permeabilization of SH‐SY5Y Cells

Plasma membranes of SH‐SY5Y were transiently permeabilized with the mild detergent digitonin. The optimum concentration of digitonin of 30 μg/mL was previously determined as the minimal effective concentration able to reduce the oxygen consumption measured by HRR upon detergent titration. To verify the integrity of mitochondrial membranes after digitonin treatment, the CytC test was performed by externally adding the CytC at the concentration of 10 μM into the cuvette and monitoring any change in the oxygen consumption rate (Doerrier et al. 2018). At least *n* = 4 independent experiments for each analysis were performed.

### 4.13 Statistical Analysis

Data are expressed as a mean or median ± SEM or SD and statistically analyzed by Student's *t* test or one‐way ANOVA followed by Tukey's test using Prism software (version 9, GraphPad Software, Boston, MA, USA). The values of *p < 0.05, **p < 0.01, and ***p < 0.001 were taken as significant.

## Author Contributions

F.C. performed cell culture maintenance, viability assays, respirometric experiments, and participated in electrophysiological experiments. S.C.N. performed VDAC1 expression and purification, chromatography, electrophysiological experiments, and contributed to the manuscript preparation. S.A.M.C. performed cell permeabilization and contributed to VDAC1 purification, electrophysiology, and respirometric experiments. P.C. and I.R.I. participated in neuroblastoma cells maintenance, cell viability, and respirometric experiments. S.Z. participated in primary cortical neurons preparation. S.R. and V.D.P. analyzed and discussed the electrophysiological results and revised the manuscript. A. Messina conceived the idea, supervised the work, and revised the manuscript. M.L.G. performed preparation of oligomers and neuron primary culture from rat embryos, discussed the data, supervised the experiments, and revised the manuscript. A. Magrì conceived the idea, conceptualized, designed, and supervised the study, analyzed and interpreted the data, provided the financial support, drew the pictures, wrote the original draft, revised, and finalized the manuscript.

## Conflicts of Interest

The authors declare no conflicts of interest.

## Supporting information


Figure S1.

## Data Availability

All data generated or analyzed during this study are included in this article and in Supporting Information.
