# Supplementary material for: VDAC1‐Targeted NHK1 Peptide Recovers Mitochondrial Dysfunction Counteracting Amyloid‐β Oligomers Toxicity in Alzheimer's Disease
Source: Aging Cell. 2025 Apr 13;24(7):e70069. doi: 10.1111/acel.70069 (PMC12266788; doi:10.1111/acel.70069)
Supplement: Supplementary file 1 — Figure S1. [file ACEL-24-e70069-s001.docx]

Supporting Information for:

**VDAC1-targeted NHK1 peptide recovers mitochondrial dysfunction counteracting amyloid-β oligomers toxicity in Alzheimer’s disease**

Fabrizio Cavallaro^1†^, Stefano Conti Nibali^1†^, Salvatore Antonio Maria Cubisino^1^, Pietro Caruso^1^,

Stefania Zimbone^2^, Iolanda Rita Infantino^1^, Simona Reina^1,3^, Vito De Pinto^1,3^, Angela Messina^3,4^,

Maria Laura Giuffrida^2^, Andrea Magrì^3,4*^

1 Department of Biomedical and Biotechnological Sciences, University of Catania, 95123 Catania, Italy

2 Institute of Crystallography, National Research Council (CNR-IC), 95126 Catania, Italy

3 we.MitoBiotech s.r.l., 95125 Catania, Italy

4 Department of Biological, Geological, Environmental Sciences, University of Catania, 95123 Catania, Italy

† These authors have equally contributed to this work

* Corresponding author

Correspondence to:

Andrea Magrì

Dept. of Biological, Geological, Environmental Sciences

University of Catania, Torre Biologica, Via Santa Sofia, 89

95123 Catania, Italy

+39 095 4781480

andrea.magri@unict.it

**Figure S1**

**
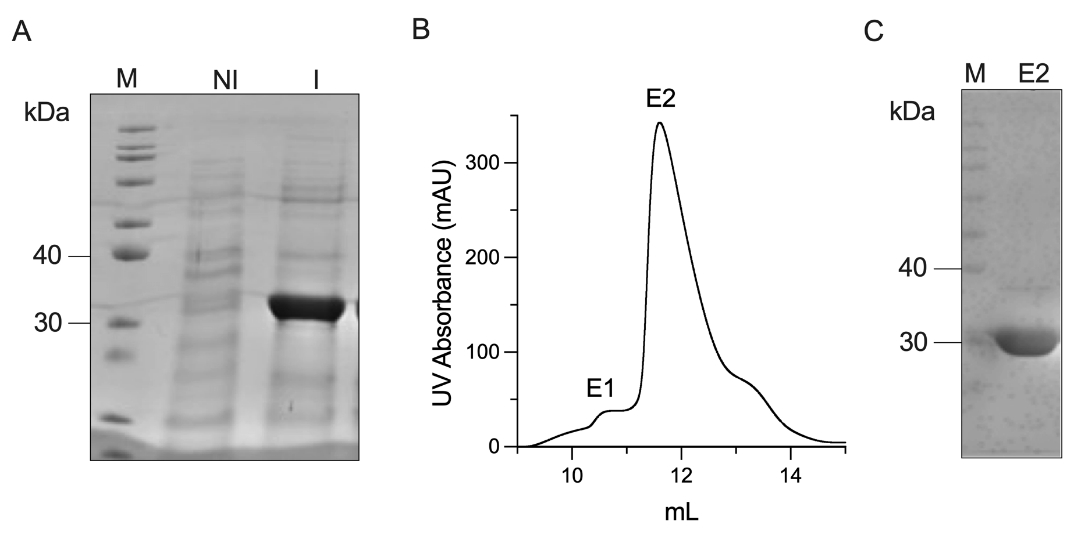
**

**Fig. S1. Expression and purification of recombinant VDAC1.**

A) SDS/PAGE analysis of the whole lysates of *E. coli* BL21 (DE3) cells transformed with the pET-21b vector carrying the encoding sequence of human VDAC1, before (NI, not induced) and after the addition of 1 mM IPTG (I, induced). M indicates the marker of molecular weights. B) Elution profile of refolded VDAC1 obtained by size‐exclusion chromatography. E1 indicates the peak corresponding to elution containing the aggregated proteins while E2 indicates the peak corresponding to elution containing the monomeric protein used in this work. C) SDS/PAGE analysis of E2 showing the level of purity of recombinant VDAC1. M indicates the marker of molecular weights.

**Figure S2**

**
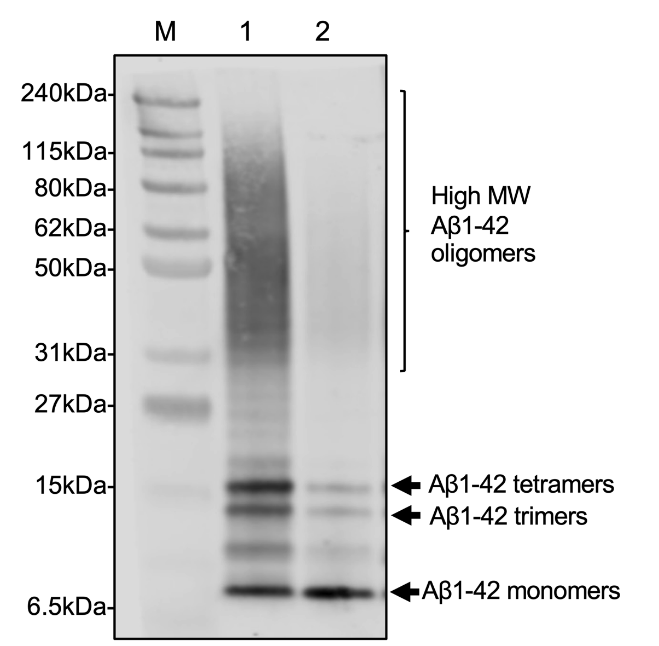
**

**Fig. S2. Preparation of Aβ_1-42_ oligomers**

Representative Western blot analysis of Aβ oligomers’ preparation. Freshly solubilized Aβ monomers were used as a control. 15μL of each solution was separated onto a 4−12% bis·tris SDS-PAGE gel and blotted with anti-Aβ N-terminal 1−16 mouse monoclonal antibody 6E10 (1:1000). M, molecular weight marker; 1, Aβ_1-42_ oligomers; 2, Aβ_1-42_ monomers.

**Figure S3**

**
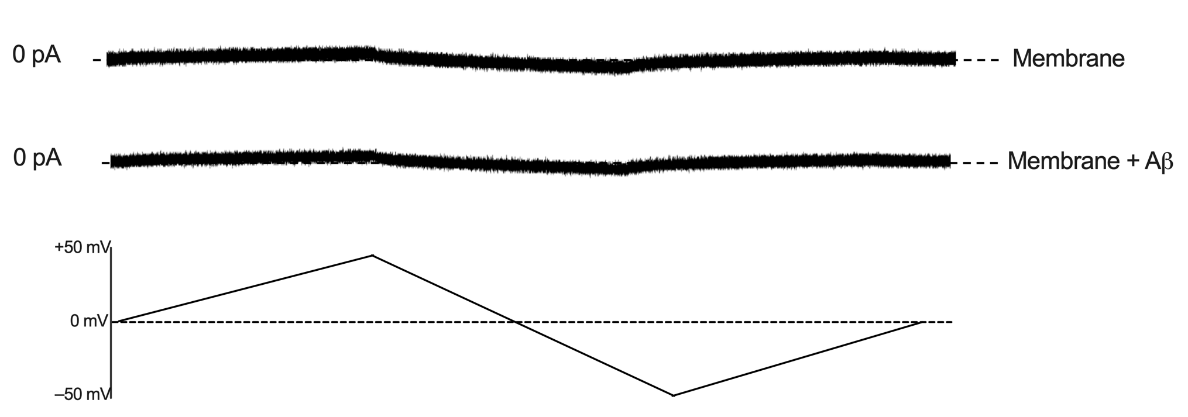
**

**Fig. S3. Evaluation of the effect of Aβ oligomers on membrane stability by triangular voltage ramps.** Representative current traces of the bilayer membrane tested before (upper trace) and after the addition of Aβ oligomers at the final concentration of 0.3 µM to either *cis* and *trans* side of the cuvette (lower trace). The stability of the current signals was monitored upon application of a voltage ramp of ± 50 mV amplitude. Experiments iwere performed in KCl buffer (1 M KCl, 1 mM Hepes, pH 7.4).

**
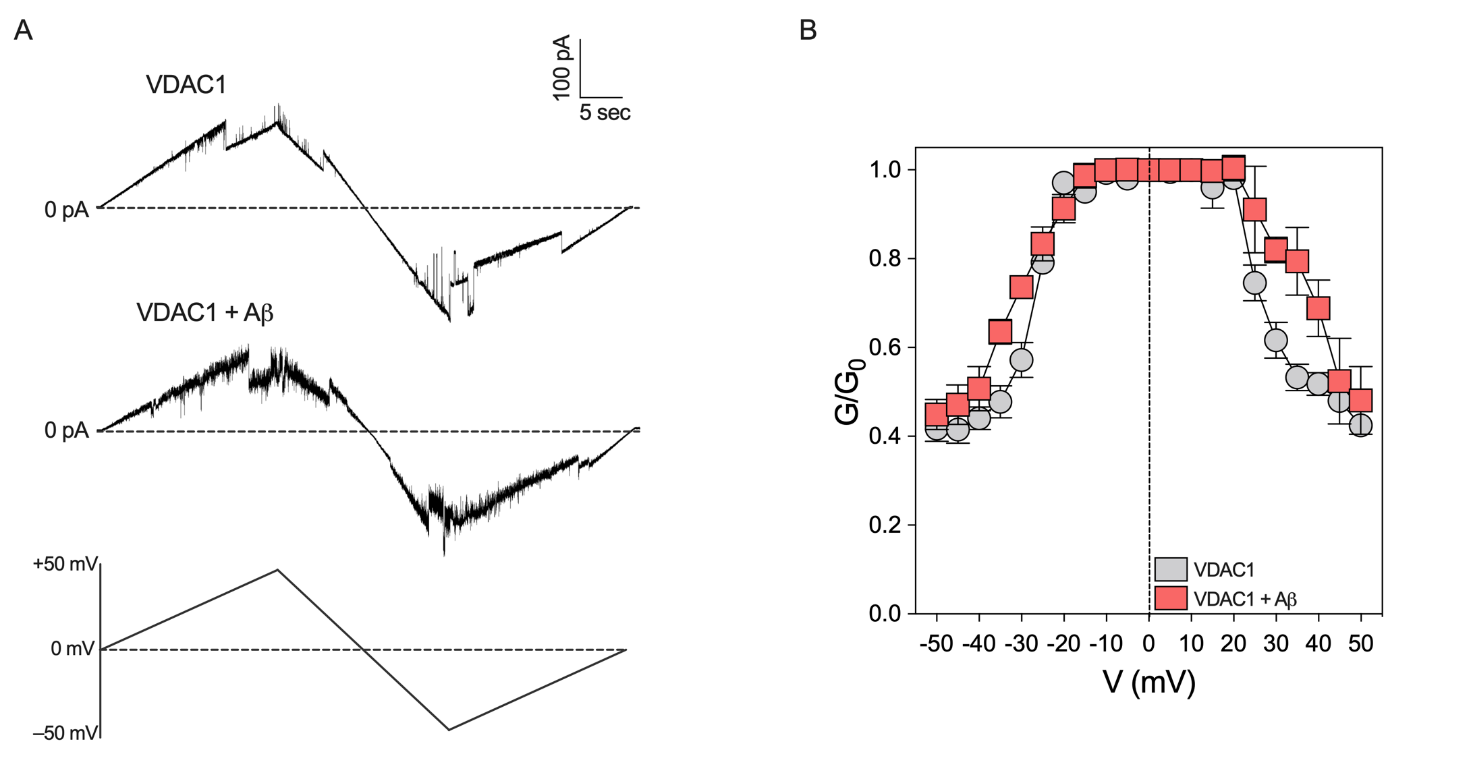
Figure S4**

**Fig. S4. Analysis of the voltage-dependency of VDAC1.**

A) Representative traces of n=3 independent experiment of VDAC1 achieved at the PLB upon the application of a triangular voltage ramps with an amplitude of ± 50 mV in 1 M KCl buffer. The traces were obtained before (upper trace) and after the addition of Aβ oligomers at the final concentration of 0.3 µM (lower trace) to either *cis* and *trans* side of the membrane. The traces were employed to calculate I/V plots. B) The bell-shaped plots of normalized average conductance as functions of the applied voltage. G/G_0_ is the normalized conductance where G_0_ is the maximal conductance. Data are expressed as a mean ± SD of n=3 independent experiments.

**Figure S5**

**
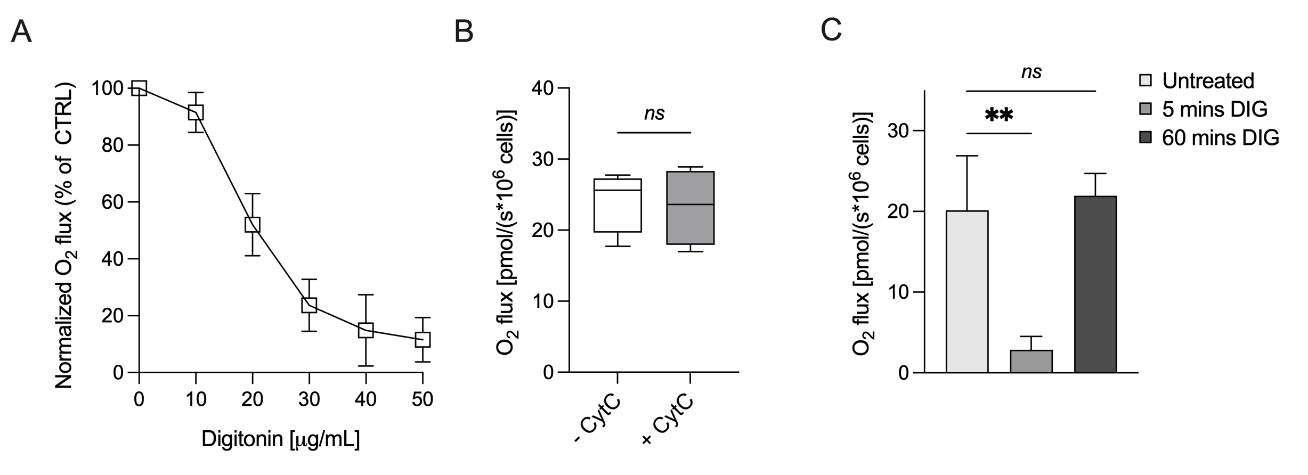
**

**Fig. S5. Determination of conditions for the permeabilization of SH-SY5Y plasma membranes.**

A) Determination of the digitonin concentration for permeabilization of plasma membranes of SH-SY5Y assayed by HRR. The reduction of Routine state was monitored upon digitonin titration and is due to the loss of the endogenous substrates through permeabilized membranes. The final concentration of digitonin was determined as the minimum concentration able to maximal reduce the oxygen consumption. Data are expressed as a mean ± SEM of n=3 independent experiments. B) Quantitative analysis of the oxygen consumption rate of permeabilized SH-SY5Y with 30 µg/mL of digitonin, measured upon the presence of succinate, before and after the addition of cytochrome c (CytC). Data are expressed as pmol/second per million cells and as a median ± SEM of n=4 independent experiments. Data were statistically analyzed by unpaired t-test; ns, not significant. C) Quantitative analysis of the oxygen consumption rate of SH-SY5Y relative to Routine state before and after the treatment with 30 µg/mL of digitonin. Respiration was assayed immediately after the digitonin treatment (5 mins) and after 60 mins from digitonin removal. Data are expressed as means ± SEM of n=3 independent experiments and analyzed by one-way ANOVA, with ** p < 0.01; ns, not significant.

**Figure S6**

**
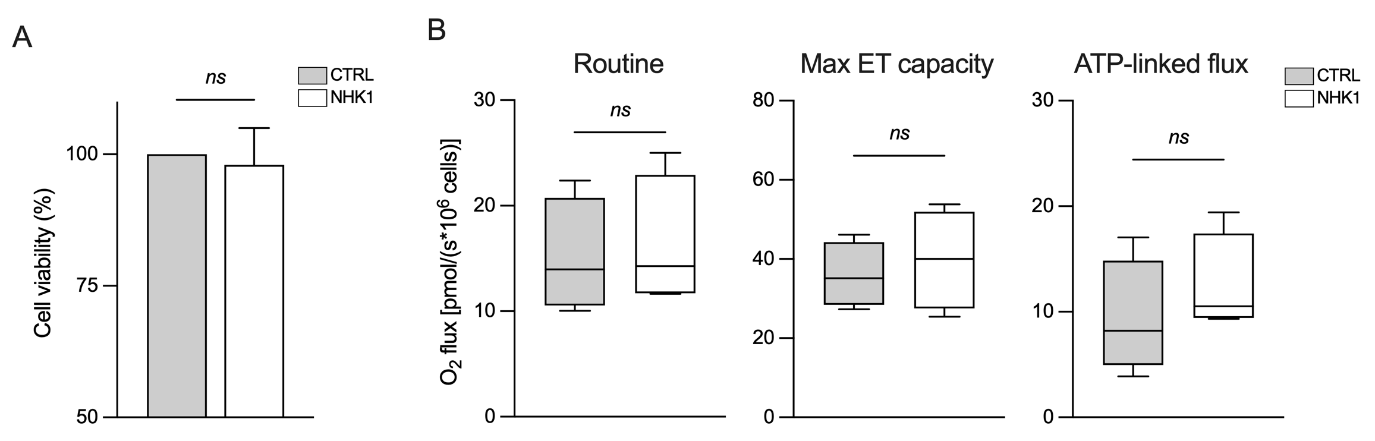
**

**Fig. S6. Analysis of NHK1 treatment on permeabilized SH-SY5Y cells.**

A) Analysis of cell viability by Trypan blue exclusion assay performed in transiently permeabilized SH-SY5Y cells exposed to NHK1 peptide at the final concentration of 10 µg/mL for 1 hour. Data are expressed as the percentage of untreated cells (control) and as a means ± SEM of n=4 independent experiments. Data were statistically analyzed by unpaired t-test; ns, not significant. B) Quantitative analysis of the oxygen consumption rates relative to Routine, maximal ET capacity and ATP-linked flux by HRR of permeabilized SH-SY5Y cells treated as in A. Data are expressed as pmol/second per million cells and as a median ± SEM of n=4 independent experiments. Data were statistically analyzed by unpaired t-test; ns, not significant.
